# Supplementary material for: The Nurse’s Role in Educating Pediatric Patients on Correct Inhaler Technique: An Interventional Study
Source: Int J Environ Res Public Health. 2022 Apr 6;19(7):4405. doi: 10.3390/ijerph19074405 (PMC8998829; doi:10.3390/ijerph19074405)
Supplement: Supplementary file 1 [file ijerph-19-04405-s001.zip › ijerph-1651455-supplementary.pdf]

Supplementary Table S1: Description of the variables included in the study based on the inhalation device used.

Inhalation device		Chamber and Mask N(%)	Chamber and Mouthpiece N(%)	Accuhaler® N(%)	Turbuhaler® N(%)	Novolizer® N(%)	Total N(%)
**Age**							
	0-4 years old	80 (66,1)	39 (32,2)	4 (3,3)	0 (0,0)	0 (0,0)	121 (30,8)
	5-9 years old	7 (4,7)	140 (94,0)	4 (2,7)	9 (6,0)	0 (0,0)	149 (37,9)
	10-15 years old	0 (0,0)	66 (53,7)	22 (17,9)	68 (55,3)	12 (9,8)	123 (31,3)
**Gender**							
	Male	68 (61,3)	106 (60,9)	20 (66,7)	36 (54,5)	6 (50,0)	236 (60,1)
	Female	43 (38,7)	68 (39,1)	10 (33,3)	30 (45,5)	6 (50,0)	157 (39,9)
**Pathologies**	Asthma	0 (0,0)	32 (18,4)	8 (26,7)	3 (4,5)	0 (0,0)	43 (10,9)
	Allergic asthma	2 (1,8)	87 (50,0)	18 (60,0)	42 (63,6)	12 (100,0)	161 (41,0)
	Recurrent wheezes	101 (91,0)	37 (21,3)	4 (13,3)	9 (13,6)	0 (0,0)	151 (38,4)
	Bronchopulmonary dysplasia	8 (7,2)	0 (0,0)	0 (0,0)	0 (0,0)	0 (0,0)	8 (2,0)
	Premature	2 (1,8)	5 (2,9)	0 (0,0)	0 (0,0)	0 (0,0)	7 (1,8)
	Laryngitis	2 (1,8)	0 (0,0)	0 (0,0)	0 (0,0)	2 (16,7)	4 (1,0)
	Cough	0 (0,0)	8 (4,6)	0 (0,0)	6 (9,1)	0 (0,0)	14 (3,6)
	Bronchiectasis	0 (0,0)	2 (1.1)	0 (0,0)	6 (9,1)	0 (0,0)	8 (2,0)
	Dyspnoea	0 (0,0)	5 (2,9)	0 (0,0)	3 (4,5)	0 (0,0)	8 (2,0)

**Pharmacotherapy**	Long-acting beta-2 agonist	12 (10,8)	35 (20,1)	8 (26,7)	6 (9,1)	6 (50,0)	67 (17,0)
	Inhaled corticosteroid	32 (28,8)	55 (31,6)	0 (0,0)	0 (0,0)	0 (0,0)	87 (22,1)
	Combined: beta-2+ corticosteroid	14 (12,6)	84 (48,3)	28 (93,3)	15 (22,7)	12 (100,0)	153 (38,9)
	Antibiotics	2 (1,8)	0 (0,0)	2 (6,7)	0 (0,0)	0 (0,0)	4 (1,0)
	Vitamin D	0 (0,0)	0 (0,0)	0 (0,0)	24 (36,4)	0 (0,0)	24 (6,1)
	Montelukast	30 (27,0)	62 (35,6)	21 (31,8)	21 (31,8)	0 (0,0)	117 (29,8)
**First visit?**							
	Yes	31 (27,9)	0 (0,0)	2 (6,7)	3 (4,5)	0 (0,0)	36 (9,2)
	No	80 (72,1)	174 (100,0)	28 (93,3)	63 (95,5)	12 (100,0)	357 (90,8)
**Degree of control of the disease****							
**Beginning**	Well Controlled	74 (66,7)	138 (79,3)	26 (86,7)	60 (90,9)	10 (83,3)	308 (78,4)
	Partially controlled	31 (27,9)	28 (16,1)	2 (6,7)	6 (9,1)	2 (16,7)	69 (17,6)
	Poorly controlled	6 (5,4)	8 (4,6)	2 (6,7)	0 (0,0)	0 (0,0)	16 (4,1)
**End**	Well controlled	72 (64,9)	150 (86,2)	26 (86,7)	48 (72,7)	12 (100,0)	308 (78,4)

	Partially controlled	34 (30,6)	21 (12,1)	4 (13,3)	18 (27,3)	0 (0,0)	77 (19,6)
**Exacerbations**	Beginning	2.1 (1.5)<br>[1,8;2,4]	1.8 (1.2)<br>[1,7;2,0]	1.9 (1.1)<br>[1,5;2,3]	1.6 (1.1)<br>[1,3;1,8]	1.3 (0.5)<br>[1,0;1,7]	1.84 (1.26)<br>[1,72;1,97]
	End	1.8 (1.4)<br>[1,5;2,1]	1.4 (1.2)<br>[1,2;1,5]	1.1 (0.9)<br>[0,7;1,4]	0.9 (1.0)<br>[0,6;1,1]	0.5 (0.5)<br>[0,2;0,8]	1.35 (1.23)<br>[1,2;1,5]
**Visit emergency care**	Beginning	0.7 (1.0)<br>[0,5;0,9]	0.7 (1.0)<br>[0,6;0,9]	0.7 (0.6)<br>[0,4;0,9]	0.5 (0.7)<br>[0,3;0,7]	0.0 (0.0)<br>[-------]	0.65 (0.92)<br>[0,55;0,74]
	End	0.6 (0.9)<br>[0,4;0,7]	0.3 (0.6)<br>[0,2;0,6]	0.3 (0.6)<br>[0,1;0,5]	0.3 (0.5)<br>[0,1;0,5]	0.0 (0.0)<br>[-------]	0.34 (0.67)<br>[0,28;0,41]
		Average age at the beginning: 2.6 (1.6)	Average age at the beginning: 7.6 (2.5)	Average age at the beginning: 9.4 (3.1)	Average age at the beginning: 11.6 (2.1)	Average age at the beginning: 10.0 (0.0)	

Supplementary Table S2: Description of the steps included in each inhalation technique according to each device studied and its percentage of correct technique.

**Inhalation Device: Chamber with Mask**	**Pre-Test N(%)**	**Post-Test N(%)**
**Step 1:** Open the inhaler, shake it upright and connect it to the chamber.	107 (96,4)	90 (81,1)
**Step 2:** Place the mask tightly around the child's mouth and nose.	109 (98,2)	105 (94,6)
**Step 3:** Press the button once with the camera in horizontal position.	105 (94,6)	107 (96,4)
**Step 4:** Maintain the position of the mask while the child breathes, observing the movement of the valve(s) (depending on whether it is uni or bivalvular).	109 (98,2)	100 (90,1)
**Step 5:** Number of inhalations: 4 inhalations are usually sufficient.	105 (94,6)	92 (82,9)
**Step 6**: Repeat the procedure for each dose with intervals of 30 seconds to 1 minute between doses.	54 (48,6)	101 (91,0)
	79 (71,2)	98 (88,3)

**Step 7**: Remove the inhaler, cap it and rinse your mouth.		

**Inhalation Device: Chamber with Mouthpiece**	**Pre-Test N(%)**	**Post-Test N(%)**
**Step 1:** Assemble the camera parts.	174 (100,0)	174 (100,0)
**Step 2:**Uncap the inhaler, shake it upright.	159 (91,4)	162 (93,1)
**Step 3:** Attach the inhaler to the chamber.	167 (96,0)	172 (98,9)
**Step 4:** Blow air out of the lungs (blow out).	164 (94,3)	165 (94,8)
**Step 5:** Place the mouthpiece of the camera in your mouth, closing your lips well and press the button, just once, with camera horizontal.	162 (93,1)	157 (90,2)
**Step 6**: Breathe in slowly, smoothly and deeply for about 5 seconds, hold your breath for about 10 seconds and exhale slowly. Repeat this step 2-5 times.	114 (65,5)	159 (91.4)
**Step 7**: If you need new doses, re-shake each time and repeat all steps with an interval of 30 seconds to 1 minute between each dose.	104 (59,8)	131 (75,3)
**Step 8**: Remove the inhaler from the chamber, cap it and rinse your mouth.	128 (73,6)	133 (76,4)
**Inhalation device: Accuhaler®**	**Pre-Test N(%)**	**Post-Test N(%)**
**Step 1:** Open the device by pushing the notch with your finger as far as it will go.	30 (100,0)	30 (100,0)
**Step 2:** Load the dose by sliding the lever or trigger that is revealed, until you hear a "click". Do not move the lever again.	30 (100,0)	30 (100,0)
**Step 3:**Expel air from the lungs by keeping the inhaler away from the mouth.	28 (93,3)	30 (100,0)
**Step 4:** Place the mouthpiece of the inhaler in your mouth, pressing it firmly with your lips, and take a few seconds to breathe in deeply.	24 (80,0)	26 (86,7)
**Step 5:** Remove the inhaler from your mouth, hold your breath for about 10 seconds, and then exhale slowly,	22 (73,3)	30 (100,0)
**Step 6**: Close the inhaler by moving the notch with your finger to the starting position.	24 (80,0)	30(100,0)

**Step 7**: If you need new doses, repeat the procedure with an interval of 30 seconds to 1 minute between every two doses.	28 (93,3)	30 (100,0)
**Step 8**:Rinse your mouth at the end and store the inhaler in a dry place.	16 (53,3)	22 (73,3)

**Inhalation device: Turbuhaler®**	**Pre-Test N(%)**	**Post-Test N(%)**
**Step 1:** Unscrew the cap and hold the inhaler in an upright position, with the thread down.	54 (81,8)	54 (81,8)
**Step 2:** Load the dose, keeping the inhaler vertical, turning the thread to the right and then to the right and then to the left until you hear a "click".	54 (81,8)	54 (81,8)
**Step 3:** Expel the air from the lungs keeping the inhaler away from the mouth.	63 (95,5)	60 (90,9)
**Step 4:** Fit the mouthpiece between your lips, holding the inhaler by threaded area, without blocking any of the inhaler's holes, and take a few seconds to breathe in deeply.	57 (86,4)	63 (95,5)
**Step 5:** Take the inhaler out of your mouth, hold your breath for about 10 seconds, and then exhale slowly.	45 (68,2)	60 (90,9)
**Step 6**: If you need a new dose, repeat all steps from point 2 with an interval of 30 seconds to 1 minute between each dose.	54 (81,8)	66 (100,0)
**Step 7**: Rinse the mouth when finished, cover the inhaler and store it in a dry place.	51 (77,3)	63 (95,5)

**Inhalation device: Novolizer®**	**Pre-Test N(%)**	**Post-Test N(%)**
**Step 1:** Uncap the inhaler.	12 (100,0)	12 (100,0)
**Step 2:** Load the dose by pressing the rear button all the way down. You can now release the button: Novolizer® is ready to be used.	10 (83,3)	12 (100,0)

**Step 3:** Expel the air from the lungs keeping the inhaler away from the mouth.	8 (66,7)	12 (100,0)
**Step 4:** Fit the mouthpiece between your lips and inhale deeply for a few seconds, continuing to inhale immediately after hearing the "click" in which the window turns from green to red. The colour change indicates that the inhalation is correct and that you are taking the medication.	10 (83,3)	10 (83,3)
**Step 5:** Take the inhaler out of your mouth, hold your breath for about 10 seconds, and then exhale slowly.	10 (83,3)	12 (100,0)
**Step 6**: If you need a new dose, wait a minimum of 30 seconds and repeat all the steps from point 2.	12 (100,0)	12 (100,0)
**Step 7**: Rinse the mouth when finished, cover the inhaler and store it in a dry place.	10 (83,3)	12 (100,0)
